# The Trimeric Autotransporter Adhesin EmaA and Infective Endocarditis †

**DOI:** 10.3390/pathogens13020099

**Published:** 2024-01-23

**Authors:** Keith P. Mintz, David R. Danforth, Teresa Ruiz

**Affiliations:** 1 Department of Microbiology and Molecular Genetics, University of Vermont, Burlington, VT 05405, USA; david.danforth@cuanschutz.edu; 2 Department of Molecular Physiology and Biophysics, University of Vermont, Burlington, VT 05405, USA; teresa.ruiz@uvm.edu

**Keywords:** bacterial adhesin, collagen interactions, electron tomography, 3D structure, biofilm

## Abstract

Infective endocarditis (IE), a disease of the endocardial surface of the heart, is usually of bacterial origin and disproportionally affects individuals with underlying structural heart disease. Although IE is typically associated with Gram-positive bacteria, a minority of cases are caused by a group of Gram-negative species referred to as the HACEK group. These species, classically associated with the oral cavity, consist of bacteria from the genera *Haemophilus* (excluding *Haemophilus influenzae*), *Aggregatibacter*, *Cardiobacterium*, *Eikenella*, and *Kingella*. *Aggregatibacter actinomycetemcomitans*, a bacterium of the Pasteurellaceae family, is classically associated with Aggressive Periodontitis and is also concomitant with the chronic form of the disease. Bacterial colonization of the oral cavity serves as a reservoir for infection at distal body sites via hematological spreading. *A. actinomycetemcomitans* adheres to and causes disease at multiple physiologic niches using a diverse array of bacterial cell surface structures, which include both fimbrial and nonfimbrial adhesins. The nonfimbrial adhesin EmaA (extracellular matrix binding protein adhesin A), which displays sequence heterogeneity dependent on the serotype of the bacterium, has been identified as a virulence determinant in the initiation of IE. In this chapter, we will discuss the known biochemical, molecular, and structural aspects of this protein, including its interactions with extracellular matrix components and how this multifunctional adhesin may contribute to the pathogenicity of A. actinomycetemcomitans.

## 1. Infectious Endocarditis

Infective endocarditis (IE) is initiated by the exposure of the underlying extracellular matrix of the cardiac valve surface due to physiological perturbation of the valve. Damage to the endothelium and exposure of the matrix leads to the binding and activation of circulating platelets, resulting in fibrin deposition, the product of blood coagulation. In the presence of transient bacteremia, bacteria can bind to the underlying matrix proteins or platelets to ultimately form an infective mass or “vegetation” composed of serum components and bacteria, which disrupts the normal flow of the blood through the heart [1].

The majority of bacterial IE cases are attributable to Gram-positive Streptococci, Staphylococci, and Enterococci species [1]. However, in up to 6% of the cases, the HACEK group of bacteria has been identified as the causative agents [2]. These species, classically associated with the oral cavity, consist of bacteria from the genera *Haemophilus* (excluding *Haemophilus* influenzae), *Aggregatibacter*, *Cardiobacterium*, *Eikenella*, and *Kingella* [3,4]. IE caused by the HACEK group of bacteria affects younger individuals and is more likely to be community-acquired than nosocomial [2,5]. These microorganisms can be detected using modern blood culture methods [6], although they may also cause “culture-negative” endocarditis, an infection from which no organisms can be isolated [5]. Among these Gram-negative organisms, *Haemophilus* and *Aggregatibacter* were the predominant genera in causing IE [7,8].

## 2. *A. actinomycetemcomitans* Physiology

**Figure 1:**
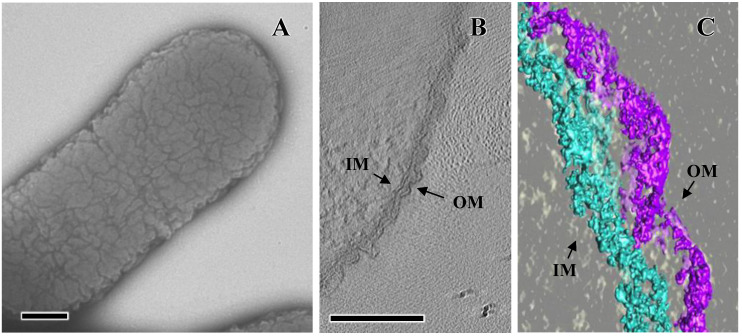
Bacterial cell surface of *A. actinomycetemcomitans*: (**A**) transmission electron micrograph of whole-mount preparations. (**B**) Central slice of a tomogram of ultrathin sections after high-pressure freezing and freeze substitution. (**C**) Segmentation of a small area of the inner (IM) and outer (OM) membranes from the tomogram. Bar, 100 nm. Adapted from [9].

*A. actinomycetemcomitans* are coccobacillus with shapes ranging from nearly cocci (0.5 µm × 0.6 µm) to bacilli (0.5 µm × 1.5 µm), depending on the culture conditions and bacterial growth phases. In contrast to most Gram-negative bacteria, which display smooth or flat outer membrane surfaces (e.g., Enterobacteriaceae), the outer membrane of the Pasteurellaceae and Moraxellaceae families displays a convoluted or corrugated morphology [9,10,11] (Figure 1A). The topography of the outer membrane of *A. actinomycetemcomitans* was described by utilizing 3D electron tomography of negatively stained bacterial preparations and using atomic force microscopy [9]. Analysis of the section profiles provided detailed information about the dimensions of the bacterial cell surface convolutions: the grooves were 12.4 ± 1.3 nm in depth and approximately 100–150 nm in diameter with a distance between grooves ranging from 65 to 165 nm. The outer membrane convolutions of *A. actinomycetemcomitans*, however, do not mirror the topography of the inner membrane, which presented a flat appearance, lacking convolutions (Figure 1B,C). The greater outer membrane surface area afforded by the convolutions may represent a selective advantage in nutrient acquisition for *A. actinomycetemcomitans* in the oral cavity. Furthermore, the dissimilarity between the inner and outer membranes may impose restrictions in the secretion and presentation of outer membrane proteins on the bacterial cell surface.

The rugose morphology is attributed to the presence of a large (141 kDa) inner membrane protein, Morphogenesis protein C (MorC), that was first identified in *A. actinomycetemcomitans* and named for its effect on the outer membrane morphology, as visualized using transmission electron microscopy [10]. The absence of MorC in the cell membrane of *A. actinomycetemcomitans* results in a bacterium with a smooth outer membrane appearance when visualized using 2D electron microscopy [9,10]. The wild-type bacterial cell exhibits a higher curvature of the outer membrane and a periplasmic space with a two-fold larger volume/area ratio when compared to the MorC mutant, as revealed using 3D electron tomography and atomic force microscopy [9]. In addition to changes in the outer membrane morphology, the inactivation of *morC* also resulted in a reduction in leukotoxin secretion in *A. actinomycetemcomitans* [10,12]. Concomitant with a reduction in leukotoxin is a reduction in cell size, an increase in autoaggregation, [10] and an increased sensitivity to membrane-destabilizing agents [13]. These pleiotropic effects are associated with changes in the abundance of multiple proteins in the membrane, including chaperones, oxidative stress response proteins, and components of the fimbrial secretion system [14]. A reduction in fimbrial subunit secretion results in a decreased number of fimbriae observed on the surface of the mutant strain and in an altered biofilm microcolony architecture [13].

In other organisms, the MorC homologs are involved in autotransporter protein incorporation into the outer membrane. However, in *A. actinomycetemcomitans*, there appears to be no impact on the autotransporter abundance in the *morC* mutant strains [12].

## 3. *A. actinomycetemcomitans* Interactions with Collagen

Bacterial colonization of the oral cavity serves as a reservoir for infection at distal body sites via hematological spreading, and poor dental health is a known risk factor for IE [15]. As stated above, IE is initiated due to the exposure of the extracellular matrix underlying the endothelium of the cardiac valve. The major component of the extracellular matrix is collagen, present in 28 different types. All collagens are composed of three polypeptide chains coiled around each other into a triple helical conformation [16]. The most abundant types of collagens include types I-III, V, and XI, which are categorized as banded or fiber-forming collagens [16]. Type IV collagen, which differs in structure from the fiber-forming collagens, is the major component of the basement membrane [16]. Less abundant non-collagenous proteins include the highly glycosylated proteins laminin and fibronectin, proteoglycans containing protein-bound glycosaminoglycan chains, and unique proteins found associated with specific tissues [17].

A common theme among both Gram-positive and Gram-negative pathogens is the ability to bind to proteins of the extracellular matrix [18]. *A. actinomycetemcomitans* has been found in the deep connective tissue, in contact with the collagen fibers of the periodontium of individuals afflicted with the aggressive form of the disease [19,20], which suggests that this bacterium interacts with collagen fibers. Studies indicate that *A. actinomycetemcomitans* binds to multiple types of immobilized, acid-solubilized collagen (types I–III and V) but not basement membrane type IV [21]. Furthermore, this organism also binds to fibronectin and laminin [21,22], additional components of the ECM.

## 4. Extracellular Matrix Protein Adhesin A (EmaA)

Bacterial outer membrane proteins were found to be essential to the interactions with the extracellular matrix [21], and several genes associated with binding to ECM proteins were identified following the screening of a transposon mutant library [21]. Disruption of a novel 5895 base pair open reading frame was identified in mutants that demonstrated a significant decrease in type V collagen binding, the collagen type found in abundance in cardiac tissue [23]. The gene product was deduced to code for a 1965 amino acid protein (202 kDa). Antibodies specific to the protein confirmed the presence of a protein of this mass associated with the outer membrane of *A. actinomycetemcomitans* [24]. The gene was designated as extracellular matrix protein adhesin A (*emaA*).

**Figure 2:**
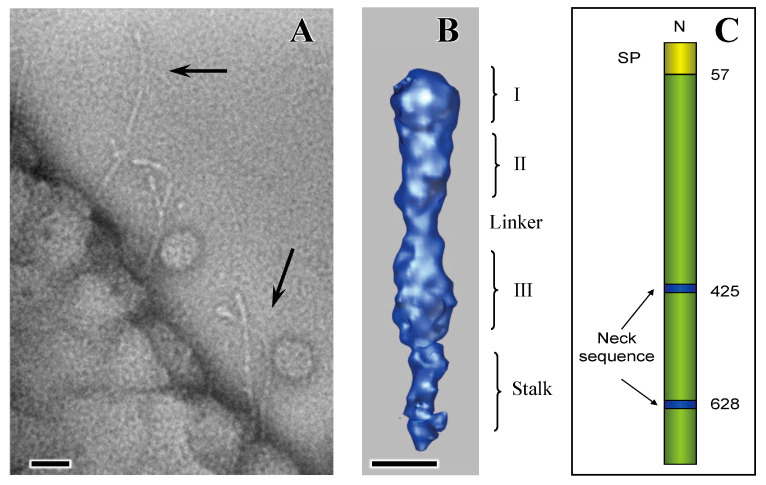
EmaA structure: (**A**) transmission electron micrograph of EmaA appendages acquired from a whole-mount preparation of a nonfimbriated bacterium showing the typical bends of the adhesin. Bar, 20 nm. Black arrows point to the most characteristic bend. (**B**) Surface representation of the 3D structure of the functional domain of EmaA obtained using electron tomography and subvolume averaging. (**C**) Cartoon with the corresponding amino acids. Only the head (57–627) and portion of the stalk domains are presented in (**B**) and (**C**). I, II and III represent subdomains SI-SIII; N: NH_2_-terminus of the polypeptide; SP: signal peptide; 57 is the start of the polypeptide after cleavage of the signal peptide; 425 and 628 represent the start amino acids of the neck sequences. Bar, 5 nm. Adapted from [35].

The EmaA protein is unique to *A. actinomycetemcomitans* [24]. However, sequence analysis suggested that EmaA belongs to a class of nonfimbrial oligomeric coiled-coil adhesins [25] or trimeric autotransporter adhesins [26], a subclass of type V secreted proteins [27] of which YadA of the *Yersinia* species is the prototypic protein. The monomer molecular mass of 202 kDa makes EmaA one of the larger members of this family of proteins, compared with YadA (42 kDa), UspA1 (83 kDa), UspA2 (60 kDa), Hia (114 kDa), and BadA (340 kDa) [28,29,30,31]. In contrast to YadA, UspA1/UspA2, and BadA, which are expressed at high densities on the bacterial surface [32], EmaA is sparsely distributed on the surface and can be more easily found at the apical end of the bacterium [33,34,35,36] (Figure 2A).

The prototypical EmaA structure consists of three identical subunits, assembled on the bacterial outer membrane, which form antenna-like structures of 3–5 nm in diameter, projecting at least 150 nm away from the bacterial surface (Figure 2A) [34]. These structures are absent in strains following the disruption of the gene sequence [34]. Three-dimensional structures of the canonical serotype b EmaA, using by 3D electron tomography and image processing, indicated that the antenna-like structure is composed of multiple domains, including an ellipsoidal-shaped head domain at the distal end of the structure, and a long stalk, which is connected to a flexible neck region [34]. The collagen-binding activity is attributed to the head domain, which corresponds to amino acids 57–627 of the monomer and encompasses the most apical 30 nm of the antennae-like structures [33,35,36]. The head domain is composed of three subdomains: a globular subdomain I (amino acids 57–225) with a diameter of ~5 nm; a cylindrical subdomain II (~4.4 × 5.8 nm, amino acids 225–433); a narrow linker with a diameter of ~3 nm; followed by another cylindrical subdomain III (~4.6 × 6.6 nm, amino acids 433–627) [33,36] (Figure 2B,C). Adjacent to the head domain is a rod-like stalk that adopts either a straight or a bent conformation at various positions along the length of the stalk structure [34,35]. The flexibility in the angular orientation of the stalk relative to the head domain is suggested to be required for optimal positioning of the functional domain to interact with collagen fibers [35].

## 5. *EmaA* Interactions with Collagen

The collagen-binding properties of EmaA were investigated utilizing acid-solubilized collagen either bound to plastic wells or embedded into an artificial basement membrane extracellular environment [37]. Since these preparations do not adequately represent the native collagen of animal tissue, isolated mouse heart valves were utilized as a representative of the in vivo conditions. The extracellular matrix (ECM) protein composition and stratification of the heart valves are conserved between humans and rabbits [38]. In this model system, both the wild-type and an isogenic *emaA* mutant bacteria had similar affinity for the tissue when the endothelium was left intact. However, following enzymatic removal of the endothelium, the mutant showed a 5–10-fold reduction in binding to the exposed underlying ECM, as compared with the wild-type bacteria. This finding indicates that EmaA plays a major role in the interaction of *A. actinomycetemcomitans* with native collagen.

The association of EmaA binding to native collagen and potentially binding to the heart valves in vivo was investigated using a well-established rabbit model for endocarditis [39]. In this model, a catheter is introduced from the carotid artery to past the aortic valve to induce minor damage in the valve tissue, resulting in the formation of sterile vegetation composed mostly of platelets and fibrin, in the absence of bacteria. Bacteria are typically injected into the animal 48 h post catheterization. Visible vegetation was formed in all three rabbits 72 h after inoculation with 1.5 or 15 × 10^7^ CFU of the bacterium. However, few, if any, bacteria were recovered from the vegetation. This is in sharp contrast to the high recovery rate typically obtained for Streptococci [40] and Staphylococci [41,42]. Taken together, these observations suggested that *A. actinomycetemcomitans* directly attaches to the damaged valve tissue rather than to the vegetation. In subsequent experiments, the rabbits were either singly or repeatedly inoculated with the bacterium at different time points either immediately or/and 48 h after catheterization [38]. The animals were euthanized ~3 h after the second inoculation and the entire aortic valves, as well as any visible vegetation, were isolated for bacterial recovery. The vegetation appeared smaller than in the prior experiment and *A. actinomycetemcomitans* was recovered, supporting the higher affinity of the bacterium for ECM molecules over the proteins composing the vegetation (e.g., fibrin).

In vivo competition studies were conducted using equal inoculum of wild-type and *emaA* isogenic mutants utilizing the modified time of inoculation in the rabbit model system. The rabbits were euthanized, and the aortic valve leaflets and any visible vegetation were removed, homogenized, and cultured onto growth media with and without the presence of antibiotics [38]. The competition index (CI) was calculated, and the value was determined to an order of magnitude less than 1 (1 indicating no difference in competitiveness between the mutant and wild-type strains). The data suggest that the *emaA* mutant colonized the traumatized heart valve approximately 10-fold less effectively than the wild-type strain, suggesting that this adhesin is a virulence determinant of *A. actinomycetemcomitans* involved in the initiation of infective endocarditis.

**Figure 3:**
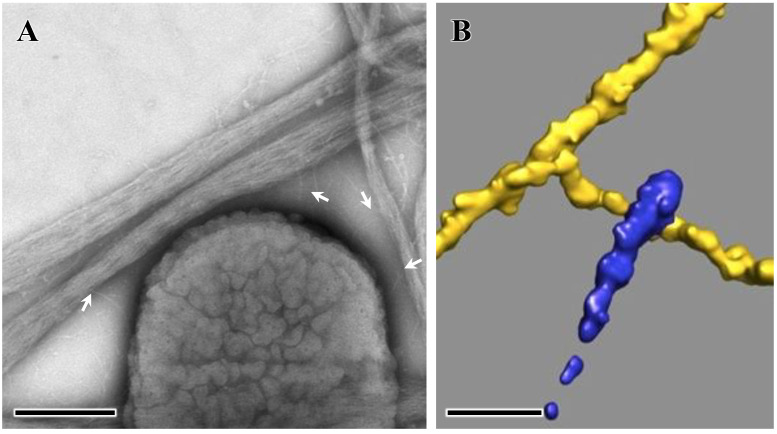
Interactions of *A. actinomycetemcomitans* with collagen visualized using electron microscopy. (**A**) Low-magnification micrograph. Arrows point to the EmaA adhesin. Bar, 0.25 nm. (**B**) Segmentation of the 3D reconstructions of the functional domain of EmaA bound to collagen. Collagen (yellow) and EmaA (blue). Bar, 10 nm. Adapted from [43].

The fine structural details of the interaction of EmaA and collagen were analyzed using 3D electron tomography and image processing techniques and using reconstituted bacterial adhesin/small collagen fiber complexes (Figure 3A) [43]. Analysis of the extracted subvolumes containing the EmaA functional domain interacting with collagen (Figure 3B) indicated that although all three subdomains (SI, SII, and SIII) of EmaA mediate the interaction, SII and SIII are more often found bound to collagen. Subdomain SII showed stronger interactions with the collagen fiber than subdomain SIII, and occasionally the tip of the apical domain SI was involved in the interactions [43]. The number of EmaA adhesins exhibiting a bend between subdomains SII and SIII (the linker region) in the bound state is higher than for the unliganded adhesin [36,43]. This bend is evocative of the one observed in the G162S EmaA substitution mutant (Subdomain SI) that could not bind collagen efficiently [33,35], which indicates that the G162S mutant adhesin is locked into a bound conformation. Furthermore, EmaA binds to collagen fibrils in a different manner than Gram-positive bacteria following either the dock, lock, and latch model [44] or the collagen hug model [45]. The EmaA/collagen interaction agrees more closely with the model proposed for the binding of YadA to collagen [46]. In this model, the interaction is governed by the electrostatic forces between the collagen fibrils and the charged residues of the trimeric YadA surface.

## 6. Secretion of EmaA and Cell Surface Expression

Proteins either targeted to the membrane or secreted into the environment are transported from the site of synthesis in the cytoplasm (by ribosomes) through the inner membrane and the periplasm and toward the outer membrane or the extracellular space. Therefore, Gram-negative bacteria utilize multiple protein secretion machineries, termed secretion systems, for transport [27,47,48]. Secretion systems are composed of protein complexes responsible for facilitating the transport of polypeptides across membranes and the periplasmic space. In the general secretory pathway, proteins are transported across the inner membrane by the Sec translocon and contain a signal peptide that indicates the protein is to be released into the periplasmic space [48]. Concomitant with translocation to the periplasm, the signal peptide is cleaved by a signal peptidase [49]. Periplasmic chaperones protect the protein from degradation on its way to the outer membrane [50].

Proteins secreted via the type V secretion system, which is dependent on the Sec translocon, encode all of the information necessary to catalyze transport across the outer membrane, giving them the name “autotransporters” [27]. This is accomplished by two main domains: the translocator domain (also known as the beta domain) and the passenger domain. The translocator domain may be composed of a single polypeptide (as is the case for monomeric autotransporters, type V_a_) or three polypeptides in the case of trimeric autotransporters, type V_c_ [51]. In both cases, the translocator domain inserts into the outer membrane and catalyzes the transport of the passenger domain through the outer membrane with assistance from the beta-barrel assembly module (BAM) complex [27,52,53]. After transport through the pore, the passenger domain is exposed to the extracellular environment. This process requires no energy and is independent of other protein factors [27].

The signal peptide of the majority of secreted proteins is found in the amino terminus of the protein. These peptides exhibit limited sequence similarity but are composed of clusters of charged or hydrophobic amino acids that are required for interaction with the protein secretory machinery in the cytoplasm [54]. Typical signal peptides are divided into three regions, containing a variable number of amino acids. An uncommon number of charged amino acids, located following the start methionine, constitute the N region, followed by a region of hydrophobic amino acids (H region) adjacent to a sequence containing the cleavage site for the inner-membrane-bound signal peptidase (C region). The later region contains small, slightly polar amino acids at the −1 and −3 positions of the signal peptide cleavage site [55].

**Figure 4:**
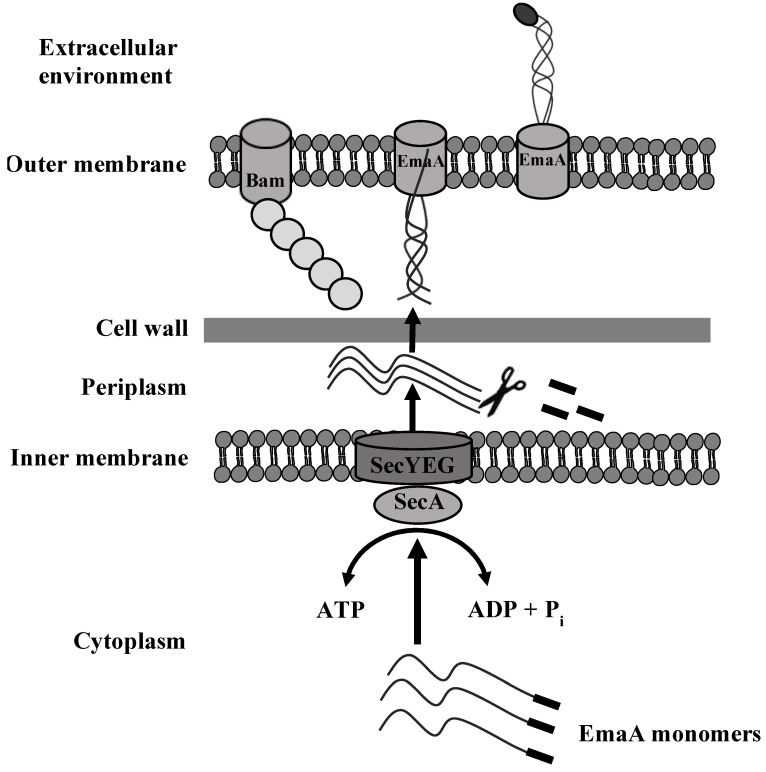
Type V secretion system of a trimeric autotransporter. Bam: β-barrel assembly machinery; EmaA: extracellular matrix protein adhesin A; SecYEG: complex of the general secretion system; SecA: ATPase motor protein associated with SecYEG.

A typical signal peptide contains between 15 and 25 amino acids [54]. However, algorithms predicted a signal peptidase cleavage site between amino acids 56 and 57 of the EmaA sequence [56]. Studies utilizing signal peptide fusion constructs with alkaline phosphatase lacking a functional signal peptide demonstrated that the first 56 amino acids acted as a signal to target the protein for translocation across the inner membrane [56] (Figure 4). Proteins containing long signal peptides are usually found in eukaryotes; however, they have also been observed in viral and other bacterial autotransporter proteins [57,58]. The individual EmaA monomers are transported to the Sec translocon via a chaperone-dependent pathway, and a specific sequence within the extended signal peptide is required for the proper secretion of EmaA at elevated temperatures that mimic the physiological temperatures the bacterium encounters during inflammation [56,59]. Following translocation and cleavage of the signal peptide, the carboxyl termini of the three EmaA monomers interact with the inner leaflet of the outer membrane and form a transmembrane pore for the presentation of an intact structure on the surface of the bacteria (Figure 4).

## 7. *A. actinomycetemcomitans* Serotypes and the Molecular Heterogeneity of EmaA

**Figure 5:**
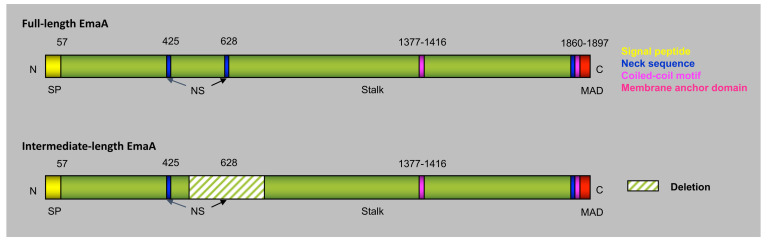
Molecular forms of EmaA proteins. Two forms of EmaA are shown: full-length and intermediate, which lacks 279 amino acids after the first neck sequence at amino acid 425. The full-length EmaA was found mainly in serotypes b and c while the intermediate EmaA was only present in serotypes d and a. Numbers correspond to amino acid number of the predicted protein. N: NH_2_-terminus of the polypeptide; SP: signal peptide; MAD: membrane anchor domain; C: COOH-terminusof the polypeptide. The whole polypeptide is represented as green with specific motifs colored as indicated in the legend.

Bacterial serotypes are dependent on the composition of the lipopolysaccharide (LPS) expressed on the surface of the bacterium. Seven serotypes (a–f) have been identified for *A. actinomycetemcomitans* [60]. Phylogenetic analysis of the *emaA* DNA sequences revealed that *A. actinomycetemcomitans* strains can be segregated cleanly into two clusters based upon serotype [61]: one cluster comprises serotypes b and c, while the remaining serotypes comprise the other [37]. Perhaps not coincidentally, EmaA is expressed as two isoforms, which are correlated with the serotype of the bacterium (Figure 5). Serotypes b and c express the cognate full-length isoform (b-EmaA, 202 kDa monomers), whereas serotypes a and d express an intermediate isoform, which is a shorter variant of EmaA containing a 279-amino-acid deletion (a-EmaA, 173 kDa monomers) [37]. Moreover, in some strains, point mutations in the DNA sequence result in truncated proteins, which are not expressed on the surface of the bacterium [37]. Both molecular isoforms of the protein (full-length and intermediate) bind to collagen [62].

**Figure 6:**
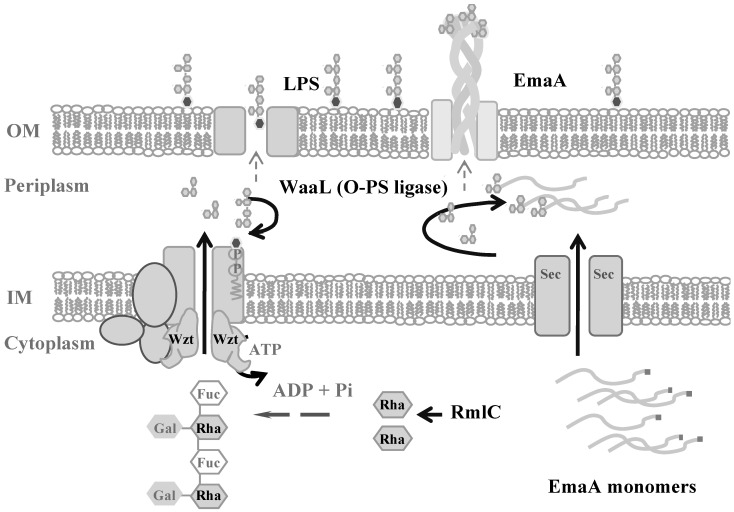
Model for the hypothetical glycosylation pathway for the trimeric autotransporter adhesin EmaA. IM: inner membrane; OM: outer membrane; LPS: lipopolysaccharide; Fuc: fucose, Rha: rhamnose; Gal: galactose; Wzt: ABC sugar transport protein; RmlC: rhamnose epimerase; WaaL: O-antigen ligase.

LPS is synthesized utilizing a well-defined sequence of enzymatic reactions, which include enzymes associated with sugar synthesis, an ABC sugar transport protein (wzt), and an O-antigen ligase (waaL) [63] (Figure 6). The enzymes in this pathway have been identified in *A. actinomycetemcomitans* [64,65,66]. Interestingly, genetic and pharmacological studies disrupting O-PS synthesis in both the serotype a and b strains revealed changes in the mass of the protein monomers (as visualized by a change in the electrophoretic mobility of the monomers) and a reduction in the amount of EmaA associated with the membrane [64]. In addition, a lectin specific to one of the serotype b O-PS sugars was demonstrated to bind to the protein [64]. These experiments suggest that: (1) EmaA is a glycoprotein modified with the sugars associated with the O-antigen and (2) EmaA utilizes the same enzymatic mechanism for post translational modification as the O-antigen does for conjugation to the LPS core oligosaccharide.

Additional experiments [67] have clearly demonstrated that *A. actinomycetemcomitans waaL* is required for the collagen-binding activity associated with EmaA and suggests that the ligase activity is important for conferring changes in the structure of this adhesin important for collagen binding.

Genetic and biochemical studies suggest that glycosylation is required for collagen binding and the stability of the protein [64,68]. A structural analysis using 3D electron tomography, iterative multireference alignment algorithms and 3D classification [62,69,70,71,72] of glycosylation-deficient mutants enables the determination of the structural role of this modification in collagen binding. The 3D structures of the functional domain of EmaA from mutant strains with glycosylation disrupted at two different stages—the *rmlc* mutant, which does not express the rhamnose epimerase, and the *waaL* mutant, which lacks the O-antigen ligase—were analyzed [70,73,74]. The structural studies of the EmaA adhesins expressed in the mutant strains suggest that glycosylation is important to maintaining the overall structural stability of the adhesin and, specifically, the proper conformation of the functional domain. Glycosylation-deficient mutant strains exhibit far fewer EmaA adhesins on the bacterial surface than the wild-type strain, which is consistent with previous protein immunoblot and mRNA expression analysis results [64,75]. In addition, the adhesins seem to “hug” the cell surface, which might ensue from modifications to the electrostatic properties of both surfaces, thus supporting YadA-like interactions with collagen. The averages from all the groups demonstrate that the mutant strain adhesins lack the three-fold symmetry characteristic of the wild-type strain and manifest a high degree of flexibility. An apparent difference between the mutant and wild-type adhesins is the overall reduced density in the structures expressed in the glycosylation mutant strains.

**Figure 7:**
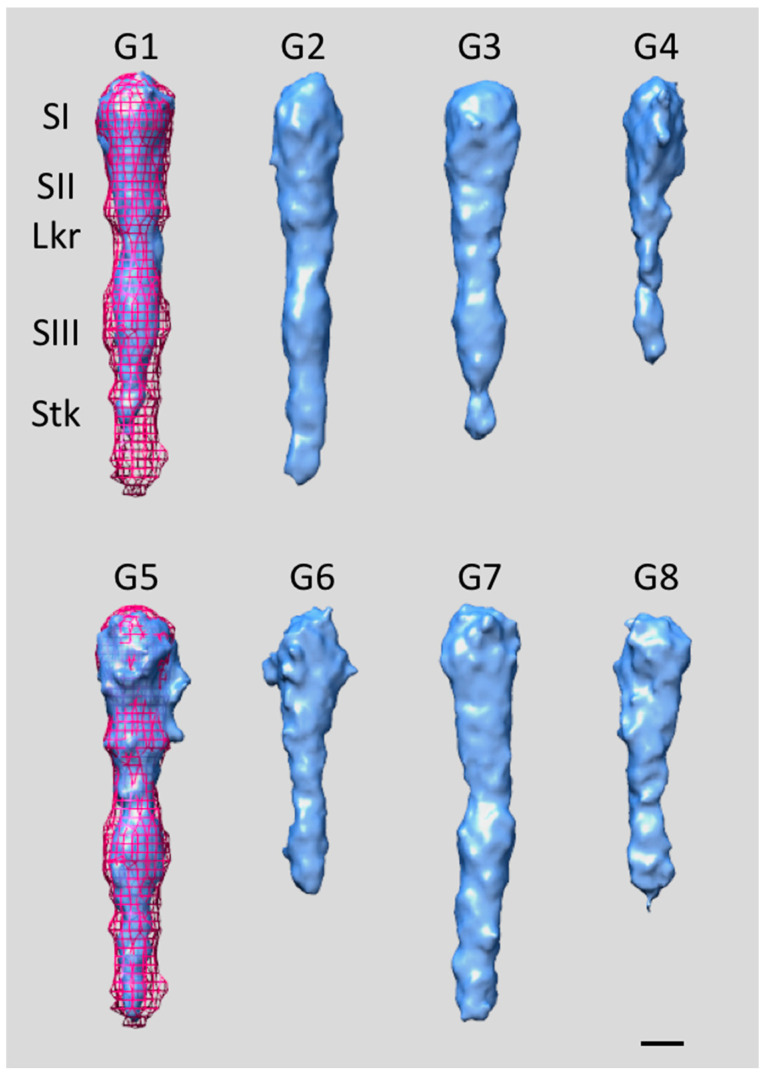
Surface representations of the EmaA averages of subgroups G1–G8 from *rmlC* mutant strains [70,74]. Mesh: wild-type EmaA structure; EmaA subdomains: SI–SIII; Lkr: linker region; Stk: stalk region. Bar, 3 nm.

**Figure 8:**
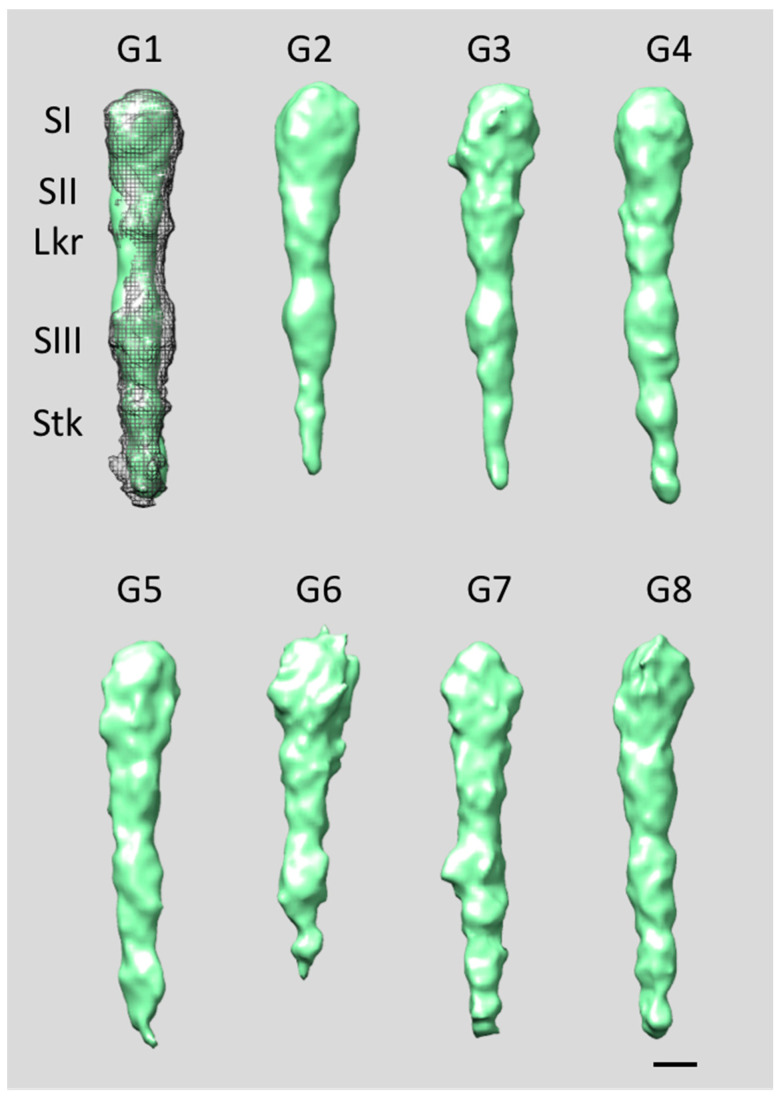
Surface representations of the EmaA averages of subgroups G1–G8 from *waaL* mutant strains [73,74]. Mesh: wild-type EmaA structure; EmaA subdomains: SI–SIII; Lkr: linker region; Stk: stalk region. Bar, 3 nm.

Subtomograms encompassing the EmaA functional domain of the *rmlc* mutant strain were separated into eight subgroups (G1–G8), with memberships ranging from 18% to 6% [70,74]. The EmaAs from this glycosylation-deficient strain exhibit reduced structural stability and clearly differ from the wild-type strain (Figure 7). Groups G4, G6, and G8 exhibit extremely low density in subdomain SIII and the stalk region, while in groups G1 and G3, the stalk is the main affected region. Only groups G2, G5, and G7 present complete functional domains comparable to those observed in wild-type EmaA [33,36,69]. However, all groups manifest a certain degree of curvature and/or bends (kinks) localized close to the linker region, either between subdomain SII and the linker or between the linker and subdomain SIII. In addition, when the structures present a complete functional domain, subdomain SIII consistently has a smaller diameter size, which can be interpreted as a reduction in either the mass or stability of the protein conformation. Similar overall characteristics were observed when analyzing subtomograms containing the EmaA functional domain of the *waaL* mutant strain, which were separated into eight subgroups (G1–G8), with memberships ranging from 25% to 6% [73,74]. With the exception of G7, all other subgroups have a strong curvature along the whole length of the functional domain (Figure 8). In most of the subgroups, subdomain SI has a larger diameter than in the wild-type strain, while the density of subdomain SIII appears weaker. In addition, a large percentage of the mutant adhesins display a strong curvature along the whole length of the functional domain and exhibit bends in places beyond the characteristic bend of the wild-type strain at the linker region [74]. The observed subtle bend between subdomains SI and SII (noticeable in G3 and G8) is reminiscent of the structural changes observed in a different G162S substitution mutant strain that exhibits greatly reduced collagen-binding activity [33,35,36,74]. Thus, the observed structural differences indicate that the lack of glycans reduces the stability of EmaA and prevents it from adopting the proper fold necessary to correctly express a functional structure capable of binding collagen. Moreover, the partial glycosylation in the *rmlC* mutant adhesins (presence of fucose) [68] has a greater impact on the structural integrity of the functional domain than the absence of ligase in the *waaL* mutant adhesins [74].

## 8. EmaA and Biofilm Formation

EmaA, originally identified as a collagen-binding adhesin, has been recently implicated in biofilm biogenesis. The absence of the protein results in strains with reduced biofilm potential, as shown in multiple fimbriated and nonfimbriated strains [76]. The lack of EmaA leads to changes in the cell density of the microcolonies formed during biofilm biogenesis, which suggests that EmaA plays an important role in mediating cell-to-cell interactions. EmaA-mediated biofilm formation is independent of the glycosylation state and the precise 3D structure of the protein, which differs from the requirements demonstrated for the collagen-binding activity of the cognate full-length isoform but more closely resembles the requirements of the shorter a-EmaA isoform [33,35,36,43]. This implies that the mechanisms governing the role of EmaA in biofilm formation and collagen binding differ [76].

Cells formed a diminished biofilm in strains lacking both fimbriae and EmaA [76]. It was hypothesized that a functional overlap or redundancy with either Aae or ApiA/Omp100 may explain these results. Epithelial cell adhesin (Aae) is a monomeric autotransporter with a mass of 130 kDa [77,78]. Whereas, ApiA/Omp100, a trimeric autotransporter with a monomeric molecular mass of 37 kDa, is a multifunctional adhesin associated with collagen binding, epithelial cell invasion, and resistance to serum killing [79,80].

The validity of the hypothesis was addressed by generating single and double mutant strains to investigate the contribution of ApiA/Omp100 and Aae to biofilm formation [81]. In the strains expressing fimbriae, the absence of ApiA/Omp100 and/or Aae did not impact biofilm formation. However, in the absence of fimbriae and EmaA, only Aae mediated biofilm formation. ApiA/Omp100 did not appear to contribute to the biofilm formation in *A. actinomycetemcomitans*. Nonetheless, when *aae* and *apiA*/*omp100* were expressed in *E. coli*, both strains demonstrated comparable biofilm formation but to a lesser degree compared with the strain expressing *emaA*. These data suggested that the contribution of EmaA and Aae to biofilm formation is highly dependent on the genetic background of the strains expressing the adhesins. The data further suggest the existence of a hierarchical functional order of these protein adhesins in biofilm formation: fimbriae (the longest of the adhesins) make primary contact with the surface, followed by the increased aggregation of bacterial cells as mediated by EmaA, culminating in more efficient adherence to the surface on the part of Aae and to a lesser extent (if any) on the part of ApiA/Omp100.

## 9. Transcriptional Control of *emaA* Expression

The adaptation of organisms to varying environmental or physiological niches is essential to survival. For the initiation of infection under the specific conditions inside a particular host niche, bacteria must adapt to the environment by reprogramming the expression of specific gene products. The environmental changes experienced by *A*. *actinomycetemcomitans* during oral infection and dissemination in the blood initiate the induction or repression of the expression of EmaA and other surface proteins. This modulation of expression is most likely part of a global regulatory reprogramming that leads to enhanced bacterial fitness for colonization of these disparate tissues.

The DNA sequence immediately upstream of the translational start site of *emaA* is sufficient for the complementation of *emaA* mutants [35,76]. This region of the DNA includes a 339 bp of the 3′ end of the CoA ligase gene based on sequence homology [24,76]. Truncation of the CoA ligase sequence, which resulted in a sequence containing only the intergenic sequence, reduced the promoter activity. This finding suggested that the regulatory elements for *emaA* expression are located within the 3′ end of the CoA ligase gene [82].

CpxR and an ArcA-binding sequence were identified using in silico analysis of the intergenic region, based on *E. coli* consensus sequences [82]. CpxR represents the response regulator for the *E. coli*/CpxAR two-component signaling system. Under stress conditions, misfolded envelope proteins accumulate, leading to the autophosphorylation of CpxA, a histidine kinase, that transfers the phosphate group to CpxR, resulting in the upregulation of a series of chaperonins and proteases that either degrade or refold the misfolded proteins, lessening envelope stress [83,84]. Furthermore, CpxR, in concert with the σ^E^ envelope stress response, contributes to the regulation of the periplasmic chaperone system [83,84,85,86]. In *E. coli*, this stress response is coupled with surface sensing and is demonstrated to control genes involved in adhesion and biofilm formation [83,87,88,89,90,91]. Thus, these systems may assist in the folding and secretion of the EmaA adhesin [82]. Over-expression of *cpxR* (in the absence of the CpxA kinase) reduces the amount of EmaA synthesized, which suggested that, at relatively high concentrations, *cpxR* downregulates EmaA expression [82]. Therefore, *cpxR* may act as a repressor instead of an activator under the growth conditions used in this study.

ArcA is the DNA-binding response regulator of the two-component regulatory system ArcAB, which regulates the adaptation of the organism to respiratory growth conditions and oxygen tension [92,93,94,95]. Under anaerobic or microaerobic respiratory conditions, ArcB, a transmembrane sensor kinase, undergoes autophosphorylation and coordinates changes in gene expression in response to changes in the respiratory and fermentative state of the cell [93,96]. The environmental niches occupied by *A. actinomycetemcomitans* during disease within or outside of the oral cavity, where EmaA is important to tissue colonization [38], reflect conditions that most likely regulate gene expression. In experiments resulting in the over-expression of the protein or genetic inactivation of *arcA*, a significant reduction in the amount of EmaA synthesized and the mass of the biofilm formed was observed [82]. Dual activation and repression by ArcA have also been reported in other bacterial species [97].

An *emaA* mutant strain, against the same fimbriated background, was observed to have a lesser effect on biofilm formation than the *arcA* mutant strain [76]. This suggests that the inactivation of *arcA* may impact other adhesins involved in biofilm formation. ArcA has been shown to regulate biofilm formation in several other species of bacteria [97,98,99]. The data suggest that ArcA acts primarily as a positive regulator of *emaA* transcription. This regulation may be mediated by either the binding of ArcA directly to the DNA or indirectly by competing with a negative regulator in response to changes in the environment.

Over-expression of two other transcriptional regulators (OxyR and DeoR) also reduced the expression of EmaA; however, little effect was detected when the same plasmids were expressed in *E. coli*. OxyR, the hydrogen peroxide stress response regulator, activates genes in the oxidative stress response system in *E. coli* [100], which regulates the surface proteins associated with an altered colony morphology and auto-aggregation [101]. OxyR is suggested to be involved in the regulation of the fimbrial secretion apparatus [102] and the autotransporter adhesin ApiA in *A. actinomycetemcomitans* [103]. DeoR regulates nucleotide catabolism and toxin production in *E. coli* [104]. The changes in *emaA* synthesis, based on excess production of specific transcription factors, do not necessarily correlate with a direct regulation by any of the proposed trans-acting regulatory elements [105]. The *A. actinomycetemcomitans* regulons are still unknown, and these proteins may interact with a large array of genes, including genes of other transcription factors, forming a network regulatory cascade that can indirectly change the expression level of a myriad of different target genes.

The minimal sequence necessary from which transcription can be initiated has been elucidated, and potential binding sites for trans-acting regulatory factors, such as CpxR and ArcA, have been deduced [82]. Interestingly, the *emaA* promoter region resembles the promoters of other major virulence adhesins of *A. actinomycetemcomitans*, including *flp*, *aae*, and *apiA* [82]. Based on these observations, it is suggested that these transcriptional regulators are involved in coordinate regulation of the adhesins required for *A. actinomycetemcomitans* colonization and pathogenesis.

## 10. Summary and Conclusions

Adhesion and tissue colonization are crucial phases during the infective process. Bacterial adhesion to extracellular matrix (ECM) proteins is a paradigm used by many pathogens for colonization and the tropism of infections. *A. actinomycetemcomitans* is typically found within the connective tissue of the periodontium in contact with collagen fibers in individuals with periodontitis [19,20,106]. The presence of bacteria in the connective tissue suggests that this organism establishes a reservoir for the continuous release of bacteria for re-infection of the gingival pocket or for the transient bacteremia responsible for systemic infections. This hypothesis is supported by the observations that the bacterium binds to the exposed underlying connective tissue of damaged heart valves and forms vegetation that alters the blood flow around the valve, leading to the development of endocarditis [5,107].

*A. actinomycetemcomitans* expresses multiple proteins associated with adhesion that are vital for colonization and contribute to its virulence [14,21,35,77,108,109]. These adhesins function hierarchically in biofilm formation: fimbriae (the longest of the adhesins) make primary contact with the surface, followed by enhanced binding and increased aggregation of the bacterial cells, as mediated by the extracellular matrix protein adhesin A (EmaA), and culminating in more specific adherence to targeted surfaces on the part of Aae and ApiA/Omp100 [76,81,110].

EmaA, the largest autotransporter protein of *A. actinomycetemcomitans*, is required for collagen binding, biofilm formation, and cell-to-cell interaction [24,36,38,76]. Three identical EmaA monomers form visible antenna-like appendages that extend 150 nm away from the bacterial surface [34,35]. The functional domain, subdivided into three subdomains (SI-SIII), is located at the distal end of the adhesin and mediates the adhesin/collagen interaction [33,35,36,43]. Moreover, EmaA is modified by a novel glycosylation mechanism involving the sugars and enzymes associated with the O-polysaccharide region of the lipopolysaccharide [64,67,68]. This post translational modification increases the stability of the adhesin and promotes a structural conformation required for collagen binding [70,73,74]. However, it does not affect other known functions [76].

## 11. Future Directions

*A. actinomycetemcomitans* expresses adhesins with molecular masses almost 20 times larger than the mass of typical bacterial proteins, suggesting that synthesis of these adhesins is regulated coordinately to manage cellular resources when their functions are not needed. Our work suggests that these adhesins are coordinately regulated by shared transcription factors [82]. To date, only a limited number of genes have been studied at the transcriptional level [111], and these studies have nearly uniformly been undertaken in highly manipulated and passaged laboratory strains that lose responsiveness during culture adaptation. A minimally manipulated strain isolated from an individual with *A. actinomycetemcomitans*-related infective endocarditis (IE) demonstrated differential regulation of *emaA* and other adhesins when grown on blood versus laboratory media, thus retaining manipulable adhesin expression). Passage of the IE strain on laboratory media reduces the surface adhesin expression, leading to an altered state, a process we refer to as transcriptional “senescence”, in which specific regulons are no longer responsive to external or intracellular signaling. This dysfunctional regulatory state has broad implications when studying gene regulation using culture-adapted laboratory strains.

This fully documented provenance and limited manipulation under well-defined environmental conditions will allow for the unique opportunity to investigate the transcriptional control of *emaA* and other virulence-related adhesins, opening up new avenues of investigation into the gene regulation and pathogenicity of this organism. Furthermore, comparison of the colonization of vastly different tissue environments by this bacterium may provide insight into the physiological changes required for this strain to transition from the oral cavity in the bloodstream into the heart.

## Data Availability

No new data were created or analyzed in this study. Data sharing is not applicable to this article.
